# Subjects’ Perception in Quantifying Printed and Digital Photos of Food Portions

**DOI:** 10.3390/nu11030501

**Published:** 2019-02-27

**Authors:** Pryscila G. Nichelle, Claudia C. B. Almeida, Suzi A. Camey, Lenine M. Garmus, Vanessa C. M. Elias, Dirce M. Marchioni, Danielle G. da Silva, Marga C. Ocke, Nadia Slimani, Regina M. Fisberg, Sandra P. Crispim

**Affiliations:** 1 Postgraduate Program in Food and Nutrition, Department of Nutrition, Federal University of Paraná (UFPR), Curitiba 80240-110, Brazil; prygharib@hotmail.com (P.G.N.); clauchoma@gmail.com (C.C.B.A.); lenine.garmus@gmail.com (L.M.G.); vah.cardozo@gmail.com (V.C.M.E.); 2 Postgraduate Program in Epidemiology, Department of Statistics, Federal University of Rio Grande do Sul (UFRGS), Porto Alegre 91509-900, Brazil; sucamey@gmail.com; 3 Department of Nutrition, School of Public Health, University of São Paulo. Research Group of Epidemiological Studies and Innovation in Food and Health—GEIAS, São Paulo 01246-000, Brazil; marchioni@usp.br; 4 Postgraduate Program in Nutrition Science, Department of Nutrition, Federal University of Sergipe, São Cristóvão 49100-000, Brazil; danygoes@yahoo.com; 5 National Institute for Public Health and the Environment, 3721 MA Bilthoven, The Netherlands; marga.ocke@rivm.nl; 6 Nutrition and Metabolism Section, International Agency for Research on Cancer, 69372 Lyon, France; slimani@iarc.fr; 7 Department of Nutrition, School of Public Health, University of São Paulo, São Paulo 01246-000, Brazil; rfisberg@usp.br

**Keywords:** food portion, photos, perception, quantification, dietary intake, adults

## Abstract

Although digital photos have the potential to improve the precision of reported portions in dietary assessment, there are few studies investigating its accuracy in comparison to printed photos. The aim of this study was to evaluate the perception of adults in quantifying food portion sizes using printed and digital photos, displayed on computer-screens and tablets. In total, 1165 evaluations were performed for 60 photos of portion sizes in Brazil. Each participant (*n* = 58) attended two sessions in the study center, with an interval of at least one week. In each session, twelve food portions were prepared and randomly evaluated by each participant in its printed and digital forms. The mean error (difference between the estimated and true portions) was not significantly different between the printed photos (2.1 g ± 47.2) and the digital ones (−6.4 g ± 53.7). The agreement on using the printed and digital photos was 91% and 90%, respectively. Furthermore, the use of the tablet was more prone to underestimation when compared to printed and computer-screen photos (*p* < 0.001). Overall, participants did not present major difficulties in perceiving the portion sizes using the printed and digital photos, but the use of tablets led to less accurate results, indicating that this needs to be further evaluated.

## 1. Introduction

A major source of error and uncertainty in the measurement of diet refers to the food portion quantification, presenting a great challenge in dietary research [1]. This hampers the determination of public policies and the establishment of health promotion actions, as well as the investigation of causal relationships between dietary factors and diseases [2,3].

Visual aids to assist individuals in estimating the amounts of foods consumed are widely used together with different self-reported dietary methods, including 24-h recall (R24h) [4,5]. The visual aids mostly used are: household measurements replicates, food models, abstract shapes, and food photos. The benefits of using these resources have been described [4,6,7,8], and in particular, the food photos are attractive, easy to be used by the interviewees, portable, low-cost and can cover a range of foods [9,10,11,12,13].

It is recognized that visual aids need to be designed specifically for each country, based on the knowledge of national food availability and ranges of intake as well as food preferences of individuals [14]. Thus, an album with photos of food portions [15] was recently developed together with the Brazilian version of the computerized R24h GloboDIET [16], which should reflect the Brazilian food consumption in view of harmonized data comparisons within Latin America and overseas. 

Furthermore, it is known that the ability of individuals in quantifying the amount of food consumed is influenced by a complex cognitive process. There are three cognitive skills that influence the estimation of the food portion sizes: (1) perception (2) conceptualization (3) and memory of consumed quantities, as defined by Nelson and colleagues [12]. Such cognitive skills are commonly evaluated in validation studies of food portion photos, from which errors are quantified [2,8,9,11,14,17]. In particular, the perception of food refers to the ability of an individual to relate to an amount of food that is shown in reality, with the amount of food shown in a visual aid [12,13].

Additionally, the use of technological innovation has been widely studied and applied in food consumption surveys [18,19]. Many of the innovations are based on the interaction of individuals with technological devices (e.g., computers.) for collecting the dietary information [18,19,20,21]. Among those, digital photos of food portions with multiple images are being used with the potential to ease the quantification by using tablets or other portable technological devices in fieldwork [18]. This may be of special importance for monitoring and epidemiological studies in which hundreds of participants need to be interviewed. In such cases, digital photos could replace printed photos, preventing the high cost printing of booklets and their transportation to different interview sites.

Although digital photos may have the potential to improve the accuracy of self-reported portion sizes, we found only one study that has compared the effectiveness of using printed versus digital photos of food portions within the same sample population [22]. This study suggested that the performance of digital and printed photos was similar, but did not explore the performance of different digital screens. Another study has described the accuracy of portion size reported in digital form compared with interviewer-administered recalls but using different visual aids [17]. The results suggested that tailoring digital images in different types and formats of foods could possibly improve their estimation. However, it highlighted the need for continued work in this aspect of dietary assessment.

Within this perspective, the present study aimed to evaluate and compare the ability of perception in adults while quantifying food portions using printed and digital photos from the Brazilian GloboDiet album.

## 2. Materials and Methods

The study was performed in the Department of Nutrition of the Federal University of Paraná (UFPR) in Curitiba, Brazil. It is part of a multicenter study, called “VALIDA”: Validation of tools to quantify the Brazilian diet. The project was approved by the Research Ethics Committee of the UFPR, number 1363816. It began in the spring of 2016 with a duration of approximately three months. In addition, written informed consent was obtained from all participants.

### 2.1. Photo Album of Food Portions and Foods Evaluated in the Study

The photo album of food portions previously developed for the Brazilian population [15] was used in the assessment. It has 96 photos, 32 new photos produced in the first half of 2015 in Brazil and 64 existing photos from the International Agency for Research on Cancer/World Health Organization (IARC/WHO) album developed in Europe. 

The foods included in the album were identified using the food consumption database obtained in the last National Food Consumption Survey (POF 2008/2009) [23]. First, variability analyses using a stepwise regression were conducted to detect the between-person variation in the intake of foods (in grams) based on selected nutrients (energy, macronutrients, vitamin A, vitamin C, iron, folate, and calcium) by region and sex [24]. Foods with over 90% accumulated *r*-square for the different nutrients were retained. Secondly, the 50 most often consumed foods were identified by region and sex. Each one of the most consumed foods was evaluated for its inclusion in the album, following the IARC guidelines (i.e., recommendations for quantifying a food with photos or other types of quantification). 

All foods in the album have a series of photos with up to six portion sizes. In this study, a total of 20 food photo series was evaluated, of which 10 were developed in Brazil: sliced mortadella, sautéed cabbage, vegetable soup with meat, jello, raw cabbage, beans, *feijoada* (Brazilian black bean recipe), cooked cassava, sliced papaya and popcorn; and 10 developed in Europe: grated carrots, rice, scrambled eggs, grilled chicken breast, apple, spaghetti, baked potato, ground meat with sauce, margarine on bread and green salad leaves. The selection of these foods was based on the following criteria: represent different food groups and formats (e.g., amorphous, liquid, solid); be frequently consumed by the local population (i.e., from Paraná), based on data of POF 2008/2009 [23] and foods with similar formats evaluated only once (e.g., boiled cassava and cassava fries). To represent the 20 food photo series, three photos of each food were randomly selected for the evaluation, representing the portion sizes small, medium and large and totalizing 60 different photos/portions.

### 2.2. Participants and Number of Evaluations

A study sample of 58 participants was included, based on the estimations suggested by Nelson and Haraldsdóttir [25] plus 20% for possible losses. For that, we considered the number of evaluations necessary to identify a minimum difference (error) of 5 g for each food with a power of 80%. The research team assumed that the 5 g minimum error was a conservative and acceptable difference for all evaluated foods in the study. As a result, we aimed at performing about 12 evaluations of food portions per participant for each type of photo: printed and digital. Out of the 60 predefined food portions, the sets of 12 evaluations were randomly allocated among the participants, meaning that by chance the participants could evaluate different portion sizes of the same food.

Recruitment of participants was conducted through direct invitation and posters placed in the university buildings. A convenience sample included subcontracted workers, technicians, lecturers or students from the university. The inclusion criterion was adults between 18 and 65 years of age. The exclusion criteria were individuals with severe visual and neurological deficits; pregnant or breastfeeding women; lecturers, technicians, and students from the field of nutrition. For checking these, a questionnaire was applied to identify the participant and his/her health condition at the beginning of the study. The recruited adults were also randomly selected according to sex and education levels. Due to the non-attendance of some participants in the second evaluation, the final number of evaluations was 1165: 630 for printed photos and 535 for digital photos.

### 2.3. Study Protocol

A pilot study with five participants was conducted before the data collection, aiming at adjusting the study protocol as well as promoting a better performance of the study design. The research team was trained to carry out the data collection (including questionnaires, forms, the collection sequence etc.), following a stepwise procedure to prevent influencing the answers from the participants. 

Participants visited the study center twice, with an interval of at least one week, to evaluate the same food for both printed and digital photos. The order of the two types of photos was allocated randomly among the participants. To prevent an effect of hungriness, participants were advised to eat within 2 h prior to the session. Similarly, foods and preparations were presented at room temperature so that their smell would not stimulate a desire for consumption. 

On the first assessment day, participants were welcomed at the university for a brief study presentation, where they were given further explanation on the study procedures. To assess the printed photos, participants were individually accompanied to the lab, where the pre-defined food portions were already weighed and properly positioned on countertops following a proposed sequence of foods. Alongside, there were the food portion photos to be used in the assessment and a form to be filled in with the participants’ answers. Everything was codified and displayed in the format of a circuit sequence. To assess the digital photos, participants were individually accompanied to another lab, where there was a sensory booth with a computer screen with a 15.0-inch computer screen with a resolution of 1024 × 768 pixels-75Hz. In addition, the evaluation of digital photos using tablets was performed using the following model: Positive brand (YPY AB10EC), which a 10.1-inch screen, 1024 × 768 pixels, format 4:3. The predefined food portions and the questionnaires were coded, and the photos on the computer or on the tablet were organized in sequence. Unlike the evaluation of printed photos, the portions were presented separately to the participants, in accordance with a predefined sequence and not in the form of a circuit. The allocation of participants who evaluated by computer or by the tablet was randomly defined. This was done with the aim of testing two types of digital screens and image quality.

For each food portion, participants had up to two minutes to choose the photo that reflected the portion displayed to him/her, resulting in a maximum of 24 min per session of 12 portions. The photos were coded by letters, from A to F, which represented the smallest to the largest portions and were presented simultaneously. Thus, the participants could choose one of the photos that best represented the food quantity. It was also possible to choose an intermediate option between them (e.g., between A and B). Furthermore, participants had to indicate whether they were familiar with the food and how often they consumed it. Consumption frequencies were: never, daily, weekly, monthly, and yearly. A total of 12 sessions with up to five participants were conducted on different days. The organization and the sequence of food portions presented in each session were randomly assigned by drawing. The completed evaluation forms were reviewed by the researchers to avoid missing information or to clarify the responses while the participants were still present. 

Foods and preparations were organized according to the pre-established photos and recipes. After the preparation, the portions could be displayed in an identical format in the photo (i.e., presentation) and using the same dish (e.g., plates and bowls) or in a different form that was displayed in the original photos (i.e., different format or dish). This was done because in reality, consumed foods by the population rarely reflect the same type of form in the photos, in terms of format and used dish. In addition, smaller or larger portions than shown in the photos were also evaluated to enable estimating intermediate portions between the photos (See supplementary data: Table S1). Foods were weighted on two types of scales: mark Explorer, capacity of 32,000 g, precision of 0.1 g and mark Katashi, capacity 3200 g and precision of 0.01 g, due to logistic constraints.

At the end of the second assessment, participants were asked to answer an evaluation questionnaire with the following questions: how the individual felt during the session (e.g., comfortable, uncomfortable, hungry, etc.); which method they preferred to quantify the food portions (printed or digital photos); and if was there any food they had more difficulty in quantifying.

### 2.4. Data Analysis

Data were doubly typed into EpiData software version 3.1 [26], with the purpose of standardizing and avoiding possible typing errors. These data were later exported to SPSS software version 22.0 [27], where statistical analyses were carried out. 

The data were analyzed as usually presented in previous similar studies as well as proposed by Nelson and Haraldsdóttir [25], comparing the results of the chosen photo by the participant and the true portion size. For that, the difference between the true and the estimated size of the portion was estimated (i.e., bias) in grams and in percentage (% error: (estimated weight-true weight/true weight) × 100). The photo series was numbered from 1 to 6, with intermediate options of 0.5 points. In this way, the differences between the amounts have been classified as follows: (a) correct choice—if the bias (difference) was 0; (b) adjacent choice was for bias between −1 and +1; (c) and the distal choice for when bias was less than −1 or greater than +1 [28]. To assess agreement, correct and adjacent portions were considered as acceptable results in the study. 

Non-parametric tests were used for the comparisons at the food level, as transformation did not improve the normality of the data distribution. The Spearman correlation was used to measure the association between the estimated amount and the true amount. Differences in grams were compared using Wilcoxon paired signed-rank test within the visual forms and between them.

In addition, the effect of personal characteristics (sex and years of education: ≤ or >12 years) as well as size (small, medium and large), presentation type (equal format and amount, different format or different amount than original photo) and consumption of the foods (yes or no) was calculated and compared between printed and digital photos. Consumption was defined as “yes” for any time consumption (daily, weekly, monthly or yearly) or “no” for never. Mann-Whiney or Kruskal-Wallis ANOVA, followed by Dunn’s post-test, was used in stratified comparisons. For all analysis, the level of significance considered was 0.05.

## 3. Results

Among the total sample, 53% were men and 76% were aged 18 to 45 years. Most of the subjects (74%) had more than 12 years of study. Moreover, 43.1% of the participants were lecturers or graduate students, 32.8% technicians and 24.1% subcontracted workers. The duration of the printed photos assessment was on average 9 min for each participant, varying from 5 to 16 min. For digital photos, the assessment lasted on average 8 min when using computer-screens (min. 5 and max. 11 min) and 11 min when using tablets (min. 5 and max. 15 min).

In the assessment of printed photos, the largest error percentage was observed for chicken (20.8%), suggesting a tendency for overestimation, while the lowest error percentage was for papaya (−0.7%). In the evaluations of the digital photos, the foods with the largest and the smallest error percentage were popcorn (39%) and beans (−0.09%), respectively (Table 1).

For printed photos, there was a statistically significant difference between the mean amount (g) of estimated portions and the true amount portions for ten of the foods: carrot, cabbage, cassava, *feijoada*, kale, margarine, jello, mortadella, popcorn, and rice. In the evaluations of digital photos, 12 foods presented statistical difference: cabbage, carrot, chicken, *feijoada*, jello, kale, margarine, mortadella, popcorn, potato, rice and soup (Table 1).

Overall, the mean error was −2.1 g ± 47.2 for printed photos (−1.1%) and −6.4 g ± 53.7 for digital ones (−3.7%), showing no statistically significant difference (*p* = 0.27). On the other hand, individual food analyses comparing printed and digital photos presented statistically significant difference for the following foods: cabbage (*p* = 0.01), cassava (*p* = 0.02), chicken (*p* = 0.02), jello (*p* = 0.01), ground beef (*p* = 0.01) and potatoes (*p* = 0.01) (Table 1).

The correlations between the true and the estimated portion sizes were larger than 0.6 for almost all foods, both in printed and digital photos (Table 1); with the exception of *feijoada* (*r* = 0.51; *p* = 0,01) in the digital photos. 

The agreement between the true and the estimated portions was 91% for the printed photos, with 36% of correct choices and 55% of adjacent choices. The food best estimated, according to this classification, was pasta with no distal choice and 63% of correct choices. Furthermore, *feijoada* had a low percentage of correct choices (20%) and a considerable percentage of choices for distal portions (17%) compared with the other foods. In the evaluations of digital photos, the concordance was 90%, with 36% correct and 54% of adjacent choices. There were no distal choices on the photo portions of ground beef, carrots, pasta and green salad leaves, which were all classified as correct or adjacent. On the other hand, the popcorn obtained the lowest number of acceptable ratings compared to other foods, with 17% of correct choices and 31% of distal choices (Table 2).

**Figure 1:**
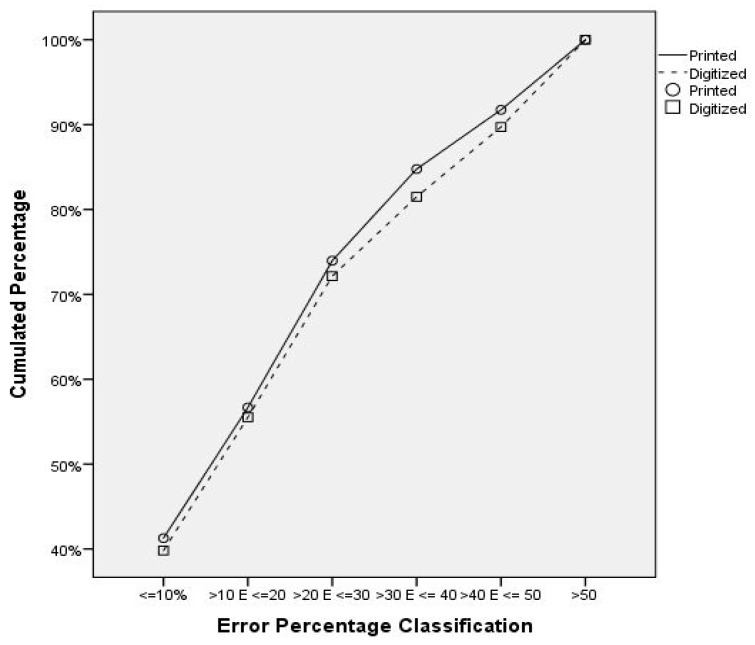
Cumulated percentage of perception assessments in relation to error percentage classification.

Overall, about 40% of the assessments presented less than 10% error, which was also similar between printed and digital photos (41.2% and 39.8%, respectively). However, this difference increased marginally when the percentage of error was larger (Figure 1). Furthermore, about 70% of the evaluations had less than 30% error.

Looking at the all samples, without food stratification, but separating the digital assessments into computer-screens and tablets, we observed that the error was significantly (*p* < 0.001) larger for tablets (mean −15 g ± 59.5) compared to printed (mean −2.1 g ± 47.2) and computer-screens (mean −0.9 g ± 47.2); results not shown in tables.

Finally, there were no differences between the mean error of groups of sex and age, within printed and digital photos (Table 3). On the other hand, participants with less than 12 years of education and that were not consumers of the food had larger errors in the evaluations of digital photos using the tablet (*p* < 0.05) when compared to more years of education and being consumers, respectively. Moreover, in the evaluations of digital photos, foods presented in a different format than the original photo had a larger bias compared to portions with an equal format, independent of the amounts. With respect to the size of the food portion, while small portions tended to be overestimated by the participants, the larger portions appeared to be underestimated (Table 3).

The majority of participants responded to “feel comfortable” during the assessments of printed (77%) and digital (73%) photos. The option of “feeling relaxed” was the second most frequent (57% for printed and 71% for digital photos). “Feeling Hungry” was a sensation reported by 17% and 7% of the sample in the evaluation of printed and digital photos, respectively.

About 38% of participants reported having had difficulty quantifying some foods in the evaluation of printed photos, especially popcorn, *feijoada* and scrambled egg. In the evaluations of digital photos, this percentage was 44%, with the most cited foods: chicken, scrambled egg and apple. The reasons for the difficulties varied. Some mentioned that was due to the dish/container being different from the photo; others due to different quantity, thickness, proportion of the food. As for the preference of the visual form of photos, 45% of participants preferred to assess the portions using printed photos, 37.5% preferred the digital photos and 17.5% were indifferent.

## 4. Discussion

The perception of individuals in quantifying foods using photos of food portions was evaluated in this study, comparing printed and digital photos. The results showed a low error in the estimates of food portions with the aid of both types of photos, with no statistical difference between the evaluation of printed and digital photos overall, although the use of the tablet showed larger underestimation when compared to the other methods of application. 

Noteworthy, specific foods, such as popcorn and *feijoada*, were not well estimated when compared to the other foods. The irregular shape of the foods and the depth of the bowls depicted in the photo can distort the concept of volume, and may therefore explain the results obtained. Particularly, amorphous foods, as *feijoada*, do not have a definite shape and will assume the format of the dish/container in which it is placed, leading to be estimated with less precision [29]. 

Furthermore, there was a tendency to underestimate most of the foods evaluated, mainly in the assessment of the digital photos. These results corroborate with other studies that have observed a tendency of food portion underestimation in their evaluations with printed photos [7,28]. For the only study comparing both printed and digital photos, no clear pattern of underestimation or overestimation was observed [22]. However, participants in the study evaluated different portions in each assessment (printed vs digital), which can hamper these comparisons.

The mean error found in the study ranged from −0.09% (black bean-digital photos) to 39% (popcorn-digital photos), with about 40% of the evaluations having less than 10% error. The minimum error found was considered smaller in comparison to other studies, such as the one from the Vereecken et al. [30] that evaluated the perception and conceptualization of 128 teenagers in Belgium and observed variation of error between −9% (potato chips and red cabbage) and −33% (peas). Likewise, de Keyzer et al. [7] evaluated the food photo perception of 111 adults in Belgium and observed that the error varied between −11.2% for bread and 197.5% for margarine. Additionally, about 70% of the present evaluations had less than 30% of error, more than what was observed in a study in Mexico [31] with 58% of evaluations within this range.

In general, the use of a tablet had a lower performance when compared to the other methods of application. The observed underestimation when using pictures displayed on the tablets could be due to the small screen size, which presented a smaller proportion of the photos as compared to the real shown portions in the printed and computer screens photos. In particular, individuals with less than 12 years of education had more difficulty in quantifying the portions using the tablet, compared to those with more than 12 years of study. Similarly, López et al. [32] assessed the use of printed photos of food portions in 76 Argentinian adults and found that the percentage of correct assessments was 60.9% in the group of people with a university degree and 52.7% in the group of people with primary education (*p* = 0.07). This may be explained by a smaller domain of the use of technological resources by individuals with low education. Preliminary results of an ongoing qualitative assessment of our study group seem to support such a statement, given the limited experience of the participants in browsing the tablet screen (unpublished results). Nevertheless, more studies are needed to evaluate this aspect in portion size estimation.

As for the size of the portions evaluated, there was a statistical difference between the small, average and large sizes, indicating the presence of the commonly known “flat slope syndrome” observed frequently in other studies [12,28,33,34]. Participants tended to overestimate small portions and to underestimate large portions. Similar to other studies, the average portion was better evaluated than the other sizes [28,35].

The way the food was presented to the participants also influenced the food portion estimation. Portions presented identically to the photos showed a smaller error than portions presented in a different format or different size. In fact, individuals tend to have more difficulty in quantifying portions that differ from the true portion of the photo, either because the distribution of the food or the used dish/container differs [11,36,37], which was corroborated by this study. It is worth noting that the studies [11,17,22,33,35,36] in this area usually differ in their methodology, making the direct comparisons of results difficult. 

The present study has some limitations. First, it should be considered that the controlled setting of the study may affect the generalization of the results. However, it is believed that the methodology applied allowed us to present interesting results on the perception of adult individuals in quantifying food portions using photos. The type of presentation that varied in both size and format is not typically approached in other studies. This is also the first study of a series that will evaluate the different cognitive abilities (i.e., conceptualization and memory) in the same population. Secondly, the small number of evaluations could result in the observed non-significant results of the study. Nonetheless, a power of more than 99% was observed when we performed post-hoc power analysis to detect the reported differences of most foods, except for papaya, beans and potato, which presented less power. But even for those cases, concordance between the correct and the reported amounts can be considered acceptable since the observed difference was less than 2 g in food amounts. 

Finally, we suggest that further studies are needed in a more realistic setting, where individuals could eat meals in a less controlled environment and dietary assessments such as 24-h recalls could be applied using the food photo booklets. Moreover, the lower performance of less educated individuals could be further explored, as for instance, through qualitative assessments. For photos that have not been well evaluated in this study (e.g., popcorn, beans), it is suggested that more evaluations are carried out for final conclusions in their use or that alternative ways of quantifications are explored.

## 5. Conclusions

The high proportion of correctly or nearly correct estimated portions indicates proper perception of the participants in using the photos in the present study, suggesting that the photos can assist in the quantification of food consumption. However, some features of the participants seemed to influence the quantification of the portion size, such as level of education. In this case, more attention should be given to the use of photos. In addition, the size and the type of food presentation also influenced quantification. Whether more sizes and food presentations should be incorporated into the album is questionable and should be explored, perhaps in digital formats where a larger range of photos is allowed. In general, there was no statistical difference between evaluations of printed and digital photos, although the evaluations using tablet resulted in worse outcomes compared to printed and computer-screens in this population, indicating that this needs to be further evaluated. 

## Figures and Tables

**Table 1:** Number of evaluations, mean and standard deviation of the true and the estimated portion sizes, error (g and %) and correlation between the true photo and the chosen one.

Printed Photos											Digital Photos										
		True Portion Size (g)		Estimated Portion Size (g)		Error (g)			% Error			True Portion Size (g)		Estimated Portion Size (g)		Error (g)			% Error		
Food	*n*	Mean	SD	Mean	SD	Mean	SD	*p*	Mean	*r* *	*n*	Mean	SD	Mean	SD	Mean	SD	*p*	Mean	*r* *	*P*
Total	631	172.6	151.9	170.6	149.5	−2.1	47.2	0.27	−1.1	0.95	535	173.0	152.5	166.6	145.6	−6.4	53.7	0.01	−3.6	0.95	0.17
Apple	36	147.4	67.6	154.6	57.2	7.1	39.1	0.37	4.8	0.76	26	152.7	66.3	150.9	55.7	−1.7	35.6	0.93	−1.1	0.75	0.09
Beans	33	188.7	118.1	191.1	127.9	2.4	32.9	0.43	1.2	0.93	28	201.0	122.8	200.7	126.4	−0.2	86.2	0.48	−0.09	0.74	0.48
Cabbage	32	64.2	45.4	59.4	35.9	−4.7	25.2	0.04	−7.3	0.82	26	64.3	45.8	54.9	32.5	−9.3	22.5	0.01	−14.4	0.86	0.01
Carrot	33	93.3	48.9	79.6	38.5	−13.7	20.5	0.001	−14.6	0.93	28	92.1	51.5	79.5	41.2	−12.5	16.6	0.001	−13.5	0.95	0.97
Cassava	33	228.5	128.6	245	122.9	16.4	37.7	0.01	7.1	0.92	27	250.3	132.2	236.4	119.1	−13.9	51.6	0.18	−5.5	0.90	0.02
Chicken	31	70.0	47.2	84.6	52.2	14.6	38.2	0.09	20.8	0.73	26	80.2	58.3	67.6	49.7	−12.5	23.1	0.01	−15.5	0.87	0.02
*Feijoada*	30	363.3	100.8	322.5	126.7	−40.8	93.3	0.02	−11.2	0.75	25	358.2	102.4	294.9	95.7	−63.2	98.2	0.01	−17.6	0.51	0.39
Green salad leaves	31	39.0	43.1	35.0	38.5	−4.0	13.1	0.16	−10.2	0.94	27	39.8	43.7	41.2	45.7	1.4	7.3	0.20	3.5	0.96	0.13
Ground beef	33	270.0	117.6	273.6	113.1	3.6	37.6	0.73	1.3	0.94	27	270.0	118.0	281.0	111.6	11.0	29.5	0.07	4.0	0.95	0.01
Jello	30	282.8	132.6	311.6	139.9	28.8	72.1	0.02	10.1	0.86	25	276.1	134.6	332.4	131.9	56.2	76.8	0.01	20.3	0.81	0.01
Kale	31	78.8	43.9	66.1	24.6	−12.7	24.4	0.02	−16.1	0.92	26	78.6	43.3	66.4	23.8	−12.2	24.4	0.03	−15.5	0.90	0.73
Margarine	30	15.8	11.4	12.9	9.03	−2.9	5.2	0.01	−18.5	0.90	29	15.8	11.6	12.6	8.8	−3.2	6.8	0.01	−20.2	0.81	0.60
Mortadella	34	20.2	4.4	22.1	4.89	1.9	3.8	0.02	9.4	0.92	24	20.7	4.5	22.9	4.0	2.2	3.1	0.004	10.6	0.94	0.08
Papaya	31	402.4	199.8	399.4	186.4	−2.9	49.9	0.79	−0.7	0.94	28	398.4	196.2	400.8	188.3	2.4	75.8	0.68	0.6	0.90	0.40
Pasta	32	212.6	157.7	202.9	145.9	−9.6	34.3	0.06	−4.5	0.95	27	195.0	158.5	193.5	154.1	−1.4	29.5	0.97	−0.7	0.95	0.64
Popcorn	28	45.4	25.7	54.9	24.2	9.4	7.4	0.001	16.2	0.95	29	44.0	24.5	61.2	22.0	17.2	11.6	0.001	39.0	0.90	0.09
Potato	34	228.5	147.4	247.0	169.5	18.5	58.6	0.08	8.0	0.94	27	222.6	153.9	195.0	134.0	−27.5	44.3	0.004	−12.3	0.97	0.01
Rice	29	256.2	108.3	235.1	89.8	−21.0	40.9	0.01	−5.2	0.91	28	260.4	108.0	230.2	74.9	−30.2	77.8	0.03	−4,2	0.64	0.07
Scrambled egg	30	159.0	93.3	137.6	53.1	−21.3	72.0	0.20	−13.3	0.68	27	151.0	91.5	145.7	59.6	−5.2	58.5	0.65	−3.4	0.83	0.22
Soup vegetables	30	306.2	117.1	290.0	114.8	−16.0	69.2	0.13	−5.2	0.68	25	302.7	117.1	275.6	125.6	−27.0	44.6	0.004	−8.9	0.80	0.97

Error (g): estimated weight − true weight.; % error: ((estimated weight − true weight)/true weight) × 100; SD = Standard Deviation; *p*: Wilcoxon paired signed-rank test for comparison between estimated and true measure (error g); * *r* = Spearman correlations; all *p* < 0.01; *P*: Wilcoxon paired signed-rank test for comparison between printed and digital photos (Mean error g).

**Table 2:** Classification of the choice of printed and digital photos in the perception of food photos study.

Food	Agreement in the Photo Choice (%)							
	Printed				Digital			
	*n*	0 Correct	<+−1 Adjacent	>+−1 Distal	*n*	0 Correct	<+−1 Adjacent	>+−1 Distal
Total	631	36	55	9	535	36	54	10
Apple	36	22	58	20	26	8	73	19
Beans	33	30	67	3	28	57	32	11
Cabbage	32	35	62	3	26	35	58	7
Carrot	33	42	52	6	28	29	71	0
Cassava	33	42	55	3	27	26	67	7
Chicken	31	23	55	22	26	27	62	11
*Feijoada*	30	20	63	17	25	16	56	28
Green salad leaves	31	39	55	6	27	48	52	0
Ground beef	33	58	39	3	27	59	41	0
Jello	30	30	60	10	25	20	60	20
Kale	31	29	68	3	26	36	62	4
Margarine	30	37	57	6	29	28	62	10
Mortadella	34	47	24	29	24	46	33	21
Papaya	31	48	48	4	28	43	46	11
Pasta	32	63	37	0	27	56	44	0
Popcorn	28	25	71	4	29	17	52	31
Potato	34	44	50	6	27	63	30	7
Rice	29	24	76	0	28	32	54	14
Scrambled egg	30	24	63	13	27	34	59	7
Soup vegetables	30	37	57	6	25	36	60	4

**Table 3:** Mean values and standard deviation of the total error of printed and digital photos, according to the characteristics of participants, portion size, type of presentation and consumption of foods (*n =* 58 participants).

Variables	Total		Printed Photos (*n*1 = 631)		Digital Photos: Computer-Screen (*n*1 = 296)		Digital Photos: Tablet (*n*1 = 239)	
	Mean of Error (g)	SD	Mean of Error (g)	SD	Mean of Error (g)	SD	Mean of Error (g)	SD
Sex								
Men	−4.3	55.1	−2.7	51.9	1.0	46.3	−17.1	72.5
Women	−3.7	44.8	−1.3	41.8	0.7	48.5	−14.1	46.4
*p*	0.85		0.67		0.40		0.91	
Age								
18–45 years old	−2.4	43.1	−0.6	39.8	0.5	47.1	−13.6	45.1
46–65 years old	−9.0	67.3	−6.8	65.9	2.7	48.4	−18.3	76.5
*p*	0.62		0.94		0.51		0.82	
Years of education								
≤12 years	−7.3	66.5	−5.9	61.7	2.4	56.4	−45.3	97.7
>12 years	−2.9	43.3	−0.9	42	−0.1	39.9	10.1	48.2
*p*	0.40		0.42		0.27		<0.001	
Portion size								
Small	11.2 ^a^	36.4	11.0 ^a^	33.1	12.2 ^a^	39	9.7 ^a^	42.6
Average	1.5 ^b^	47.1	0.3 ^b^	47.3	4.6 ^a^	49.2	1.1 ^a^	45.0
Large	−25.2 ^c^	57.8	−17.9 ^c^	54.3	−21.6 ^b^	49.9	−43.4 ^b^	66.9
*p*	0.001		0.001		<0.001		0.001	
Type of presentation								
As the original portion	−0.7	46.1	3.3	41.2	−2.8 ^a^	45.2	−9.0 ^a^	57.6
Different format	−12.9	72.3	−10.4	68.7	8.5 ^b^	54.5	−81.3 ^b^	89.2
Different amount	−4.9	43.5	−6.4	41.7	2.4 ^a^	44.8	−7.7 ^a^	41.8
*p*	0.99		0.61		0.05		0.001	
Food consumption								
Yes	−3.6	49.2	-1.9	47.3	0.2	47.4	-12.8	56.1
No	−12.3	64.7	−3.2	46.6	18.5	40.8	−51.9	89.7
*p*	0.94		0.32		0.21		0.03	

*n*1 = number of evaluations; *p*-values: Mann-Whitney or Kruskal-Wallis test followed by Dunn’s post-test. Values with different letters are statistically different between them.

## Supplementary Materials

The following are available online at https://www.mdpi.com/2072-6643/11/3/501/s1, Table S1: Description of food portions of the original photo and of the evaluated portion, according to size and format.

## References

[1] Souverein O.W., de Boer W.J., Geelen A., van der Voet H., de Vries J.H., Feinberg M., van’t Veer P. (2011). Uncertainty in intake due to portion size estimation in 24-h recalls varies between food groups. J. Nutr..

[2] Bouchoucha M., Akrout M., Bellali H., Bouchoucha R., Tarhouni F., Mansour A.B., Zouari B. (2016). Development and validation of a food photography manual, as a tool for estimation of food portion size in epidemiological dietary surveys in Tunisia. Libyan J. Med..

[3] Willett W. (2012). Nutritional Epidemiology.

[4] Guthrie H.A. (1984). Selection and quantification of typical food portions by young adults. J. Am. Diet. Assoc..

[5] Subar A.F., Kirkpatrick S.I., Mittl B., Zimmerman T.P., Thompson F.E., Bingley C., Willis G., Islam N.G., Baranowski T., McNutt S. (2012). The automated self-administered 24-hour dietary recall (asa24): A resource for researchers, clinicians, and educators from the national cancer institute. J. Acad. Nutr. Diet..

[6] Chambers E., Godwin S.L., Vecchio F.A. (2000). Cognitive strategies for reporting portion sizes using dietary recall procedures. J. Am. Diet. Assoc..

[7] De Keyzer W., Huybrechts I., De Maeyer M., Ocke M., Slimani N., van ’t Veer P., De Henauw S. (2011). Food photographs in nutritional surveillance: Errors in portion size estimation using drawings of bread and photographs of margarine and beverages consumption. Br. J. Nutr..

[8] Godwin S.L., Chambers E., Cleveland L. (2004). Accuracy of reporting dietary intake using various portion-size aids in-person and via telephone. J. Am. Diet. Assoc..

[9] Foster E., Matthews J.N., Nelson M., Harris J.M., Mathers J.C., Adamson A.J. (2006). Accuracy of estimates of food portion size using food photographs—The importance of using age-appropriate tools. Public Health Nutr..

[10] Frobisher C., Maxwell S.M. (2003). The estimation of food portion sizes: A comparison between using descriptions of portion sizes and a photographic food atlas by children and adults. J. Hum. Nutr. Diet..

[11] Lucas F., Niravong M., Villeminot S., Kaaks R., Clavelchapelon F. (1995). Estimation of food portion size using photographs—Validity, strengths, weaknesses and recommendations. J. Hum. Nutr. Diet..

[12] Nelson M., Atkinson M., Darbyshire S. (1994). Food photography. I: The perception of food portion size from photographs. Br. J. Nutr..

[13] Nelson M., Atkinson M., Darbyshire S. (1996). Food photography II: Use of food photographs for estimating portion size and the nutrient content of meals. Br. J. Nutr..

[14] Trolle E., Vandevijvere S., Ruprich J., Ege M., Dofkova M., de Boer E., Ocke M. (2013). Validation of a food quantification picture book targeting children of 0–10 years of age for pan-european and national dietary surveys. Br. J. Nutr..

[15] Crispim S.P., Fisberg R.M., Almeida C.C.B., Nicolas G., Knaze V., Pereira R.A., Marchiori D.M.L., Santos N.A.S., Steluti J., Slimani N. (2017). Manual Fotográfico De Quantificação Alimentar.

[16] Bel-Serrat S., Knaze V., Nicolas G., Marchioni D.M., Steluti J., Mendes A., Crispim S.P., Fisberg R.M., Pereira R.A., Araujo M.C. (2017). Adapting the standardised computer- and interview-based 24 h dietary recall method (GloboDIET) for dietary monitoring in Latin America. Public Health Nutr..

[17] Kirkpatrick S.I., Potischman N., Dodd K.W., Douglass D., Zimmerman T.P., Kahle L.L., Thompson F.E., George S.M., Subar A.F. (2016). The use of digital images in 24-hour recalls may lead to less misestimation of portion size compared with traditional interviewer-administered recalls. J. Nutr..

[18] Arens-Volland A.G., Spassova L., Bohn T. (2015). Promising approaches of computer-supported dietary assessment and management-current research status and available applications. Int. J. Med. Inform..

[19] Stumbo P.J. (2013). New technology in dietary assessment: A review of digital methods in improving food record accuracy. Proc. Nutr. Soc..

[20] Illner A.K., Freisling H., Boeing H., Huybrechts I., Crispim S.P., Slimani N. (2012). Review and evaluation of innovative technologies for measuring diet in nutritional epidemiology. Int. J. Epidemiol..

[21] Eldridge A.L., Piernas C., Illner A.K., Gibney M.J., Gurinovic M.A., de Vries J.H.M., Cade J.E. (2019). Evaluation of new technology-based tools for dietary intake assessment-an ilsi europe dietary intake and exposure task force evaluation. Nutrients.

[22] Timon C.M., Cooper S.E., Barker M.E., Astell A.J., Adlam T., Hwang F., Williams E.A. (2018). A comparison of food portion size estimation by older adults, young adults and nutritionists. J. Nutr. Health Aging.

[23] IBGE (2011). Pesquisa De Orçamentos Familiares 2008–2009: Análise do Consumo Alimentar Pessoal No Brasil.

[24] Molag M.L., de Vries J.H., Duif N., Ocke M.C., Dagnelie P.C., Goldbohm R.A., van’t Veer P. (2010). Selecting informative food items for compiling food-frequency questionnaires: Comparison of procedures. Br. J. Nutr..

[25] Nelson M., Haraldsdottir J. (1998). Food photographs: Practical guidelines I. Design and analysis of studies to validate portion size estimates. Public Health Nutr..

[26] Epidata D.K. 2000–2010, Version 3.1, Denmark, EpiData Association. http://www.epidata.dk/download/php..

[27] IBM Corp. (2013). IBM SPSS Statistics for Windows, Version 22.0.

[28] Naska A., Valanou E., Peppa E., Katsoulis M., Barbouni A., Trichopoulou A. (2016). Evaluation of a digital food photography atlas used as portion size measurement aid in dietary surveys in Greece. Public Health Nutr..

[29] Thoradeniya T., de Silva A., Arambepola C., Atukorala S., Lanerolle P. (2012). Portion size estimation aids for asian foods. J. Hum. Nutr. Diet..

[30] Vereecken C., Dohogne S., Covents M., Maes L. (2010). How accurate are adolescents in portion-size estimation using the computer tool young adolescents’ nutrition assessment on computer (yana-c)?. Br. J. Nutr..

[31] Bernal-Orozco M.F., Vizmanos-Lamotte B., Rodriguez-Rocha N.P., Macedo-Ojeda G., Orozco-Valerio M., Roville-Sausse F., Leon-Estrada S., Marquez-Sandoval F., Fernandez-Ballart J.D. (2013). Validation of a mexican food photograph album as a tool to visually estimate food amounts in adolescents. Br. J. Nutr..

[32] López B.L., Longo N.E., Carballido P.M., Di Carlo P. (2006). Validación del uso de modelos fotográficos para cuantificar em tamaño de las porciones de alimentos. Rev. Chil. Nutr..

[33] Huybregts L., Roberfroid D., Lachat C., Van Camp J., Kolsteren P. (2008). Validity of photographs for food portion estimation in a rural west african setting. Public Health Nutr..

[34] Szenczi-Cseh J., Horvath Z., Ambrus A. (2017). Validation of a food quantification picture book and portion sizes estimation applying perception and memory methods. Int. J. Food Sci. Nutr..

[35] Souza R.G.M.d., Campos M.I.V.A.M., Cordeiro M.d.M., Monego E.T., Peixoto M.d.R.G. (2016). Validação de fotografias de alimentos para estimativa do consumo alimentar. Rev. Nutr..

[36] Lillegaard I.T., Overby N.C., Andersen L.F. (2005). Can children and adolescents use photographs of food to estimate portion sizes?. Eur. J. Clin. Nutr..

[37] Vilela S., Lopes C., Guiomar S., Severo M., Rangelova L., Petrova S., Horvath Z., Cseh J., Schweter A., Lindtner O. (2018). Validation of a picture book to be used in a pan-european dietary survey. Public Health Nutr..

