# Supplementary material for: Subjects’ Perception in Quantifying Printed and Digital Photos of Food Portions
Source: Nutrients. 2019 Feb 27;11(3):501. doi: 10.3390/nu11030501 (PMC6471321; doi:10.3390/nu11030501)
Supplement: Supplementary file 1 [file nutrients-11-00501-s001.pdf]

Table 1. Description of food portions of the original photo and of the evaluated portion, according to size and format.

Foods	Food description	Amount of food (g) in the original photo			Amount of food (g) presented			Portion with different format than the original photo
		Small	Average	Large	Small	Average	Large	
**Apple**	**Whole unit**	66	198	217	66	178[a]	217[b]	Different Apple
Beans	With liquid	61	174	354	61	160[a]	354	-
Cabbage	Chopped, raw	17	57	123	17	51[a]	123	-
Carrot	Grated	25	85	160	37[a]	85	160	-
Cassava	Pieces, cooked	118	238	415	118	193 [a]	415	-
Egg	Scrambled	69	123	190	69[b]	123	288[a]	Position on the plate
Feijoada	In deep plate	172	376	478	236[a]	376[c]	478[c]	Different plate
Jello	Amorphous format	116	298	480	116[b]	298	434[a]	Position on the plate
Green salad leaves	Mixed green salad	5	17	100	2.5[a]	17	100	-
Ground beef	With tomato sauce	120	251	402	120[b]	288[a]	402	Position on the plate
Kale	Chopped, sautéed	34	62	90	34	62	135[a]	-
Margarine	Spreaded on sliced bread	2.5	15	22	2.5	15	30[a]	-
Mortadella	Sliced	17	19	27	17[b]	18[a]	27	Slice thickness
Papaya	Slices	139	390	614	139	438[a]	614	-
Pasta	Cooked spaghetti	61	170	400	35[a]	170	400	-
Popcorn	Glass bowl	23	41	77	17[a]	41[b]	77	Different Bowl
Potato	Boiled, diced	70	228	405	35[a]	228[b]	405	Position on the plate
Rice	White cooked	112	239	403	141[a]	239	403[b]	Position on the plate
Vegetable soup	In deep plate	96	324	400	96	362[a]	400[b]	Different plate

a The evaluated portion ranged from up to 100% smaller to 50% larger than the original portion, depending on the amount of the adjacent photo, b Portion assessed on different format that the one from the original photo, c Half of the evaluated portions had the same size as the photo and the other half had a different format
